# Poly[[μ_7_-l-cysteato(2−)]disodium]

**DOI:** 10.1107/S1600536811035525

**Published:** 2011-09-14

**Authors:** Fu-Hong Liu

**Affiliations:** a Basis Department, Jilin Business and Technology College, Hao Yue Road No. 1606, Changchun, Jilin, People’s Republic of China

## Abstract

The title compound {systematic name: poly[[μ_7_-(2*R*)-2-amino-3-sulfonato­propano­ato]disodium]}, [Na_2_(C_3_H_5_NO_5_S)]_*n*_, was obtained through solvent-thermal reaction of l-cysteic acid and aqueous sodium hydroxide. The monomer consists of two Na^+^ cations that are coordinated to the deprotonated amino acid. The latter acts as donor utilizing all available coordination sites, *viz.* the amino, the carboxyl­ate and the sulfonate residues, so producing a monomeric framework in which the two coordinated Na^+^ ions have different coordination spheres and geometries. One of the Na^+^ ions has an O_5_ coordination sphere with a typical geometric arrangement, inter­mediate between trigonal–bipyramidal and square–pyramidal; all the O atoms from the amino acid (three from the sulfonate and two from the caboxylate residues) act as donors. The second Na^+^ ion is tetracoordinated within an NO_3_ coordination sphere. The Na^+^ ion binds to the amino N atom, to one of the O atom of the carb­oxy­lic residue and to two O atoms of the sulfonate group in a distorted tetra­hedral arrangement. As the sulfonate O atoms bind to both Na^+^ ions, a three-dimensional polymeric framework is obtained.

## Related literature

For l-cysteic acid in coordination compounds, see: Hendrickson & Karle (1971 ▶); Ramanadham *et al.* (1973 ▶). For metal-organic frameworks of l-Cysteic acid, see: Bharadwaj *et al.* (1985 ▶); Riley *et al.* (2002 ▶); Huang *et al.* (2009 ▶). 
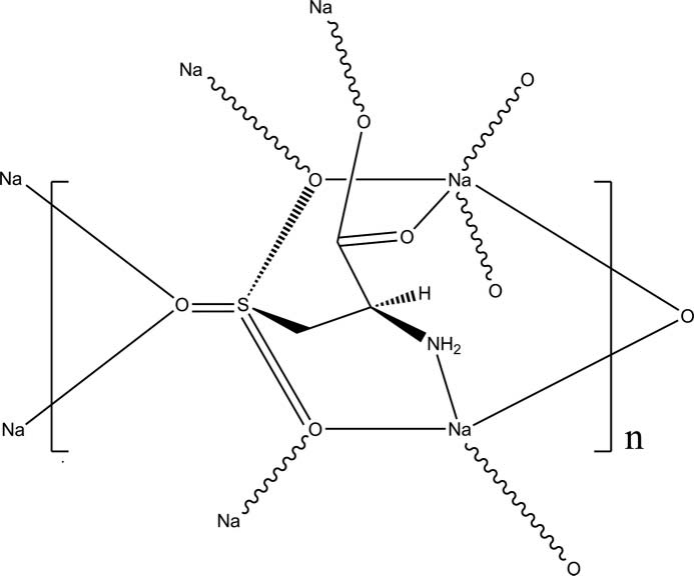


## Experimental

### 

#### Crystal data

[Na_2_(C_3_H_5_NO_5_S)]
*M* *_r_* = 213.13
Monoclinic, 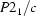

*a* = 5.7574 (12) Å
*b* = 11.875 (2) Å
*c* = 11.691 (3) Å
β = 109.15 (3)°
*V* = 755.1 (3) Å^3^
*Z* = 4
Mo *K*α radiation
μ = 0.52 mm^−1^
*T* = 298 K
0.24 × 0.22 × 0.20 mm

#### Data collection

Rigaku SCX-MINI diffractometer
Absorption correction: multi-scan (*ABSCOR*; Higashi, 1995 ▶) *T*_min_ = 0.885, *T*_max_ = 0.903
7845 measured reflections
1740 independent reflections
1463 reflections with *I* > 2σ(*I*)
*R*_int_ = 0.044

#### Refinement

*R*[*F* ^2^ > 2σ(*F* ^2^)] = 0.060
*wR*(*F* ^2^) = 0.159
*S* = 1.06
1740 reflections
109 parameters
H-atom parameters constrained
Δρ_max_ = 0.82 e Å^−3^
Δρ_min_ = −0.94 e Å^−3^

### 

Data collection: *PROCESS-AUTO* (Rigaku, 1998 ▶); cell refinement: *PROCESS-AUTO*; data reduction: *CrystalStructure* (Rigaku/MSC, 2002 ▶); program(s) used to solve structure: *SHELXS97* (Sheldrick, 2008 ▶); program(s) used to refine structure: *SHELXL97* (Sheldrick, 2008 ▶); molecular graphics: *ORTEP-3 for Windows* (Farrugia, 1997 ▶); software used to prepare material for publication: *publCIF* (Westrip, 2010 ▶).

## Supplementary Material

Crystal structure: contains datablock(s) I, global. DOI: 10.1107/S1600536811035525/go2024sup1.cif

Structure factors: contains datablock(s) I. DOI: 10.1107/S1600536811035525/go2024Isup2.hkl

Additional supplementary materials:  crystallographic information; 3D view; checkCIF report

## Figures and Tables

**Table 1:** Selected bond lengths (Å)

Na1—O1	2.478 (3)
Na1—O2^i^	2.427 (3)
Na1—O3	2.415 (4)
Na1—O4^ii^	2.351 (4)
Na1—O5^iii^	2.364 (3)
Na2—N1	2.976 (5)
Na2—O3^iv^	2.905 (5)
Na2—O4	3.028 (5)
Na2—O5^iii^	2.922 (5)

Symmetry codes: (i) 
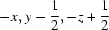
; (ii) 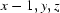; (iii) 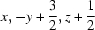; (iv) 
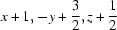
.

**Fig. 1.**
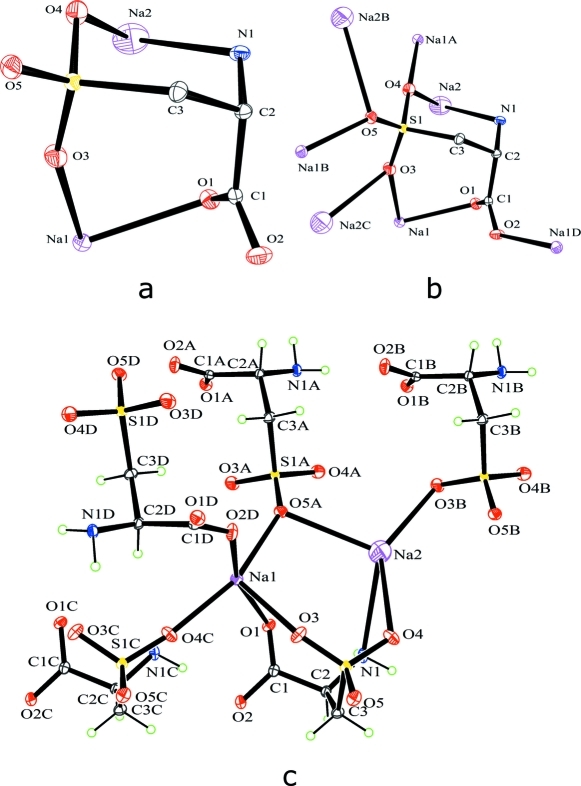
The ORTEP-3 view of the title complex with atom labels and 50% probability displacement ellipsoids for non-H atoms. a. The asymmetric unit of the title complex. b. The µ7 connectivity of each L-Cysteic acid. Other Na+ cations are hidden for breifness. c. The coordination mode for Na1 and Na2. The other L-Cysteic acid is hidden for breifness.

**Fig. 2.**
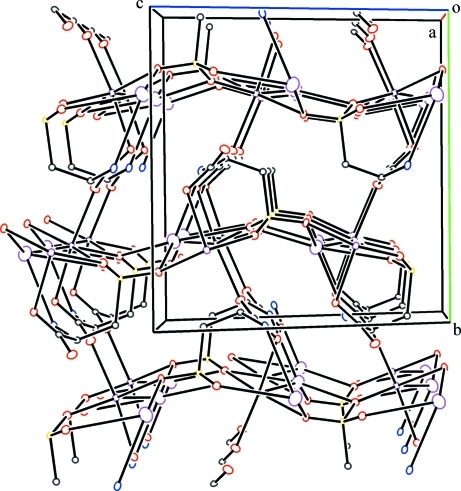
The 2x2x2 packing view of the title complex, viewed down the a axis for non-H atoms.

## supplementary crystallographic information

### Comment

Amino acids are of interest in coordination chemistry because they have a large number of highly flexible derivatives capable of forming a wide range of metal compexes. Recently, L-Cysteic acid, (Hendrickson *et al.*, 1971), (Ramanadham *et al.* 1973) has become an important ligand in the coordination and construction of metal-organic frameworks (MOFs), as a result, some of these frameworks with unusual topologies have been reported by (Bharadwaj *et al.*, 1985, Riley *et al.*, 2002, and Huang *et al.* 2009). As a part of our work in amino acid coordination research we chose L-Cysteic acid as our ligand. The title compound has recently been prepared in our laboratory and its structure is reported here.

The molecular structure of [C_3_H_5_NNa_2_O_5_S]_n_ is presented in Fig.1a-c. Fig.1a shows that two Na^+^ cations are coordinated to one L-Cysteic acid while Fig.1b shows the µ7 connectivity of each L-Cysteic acid. Each L-Cysteic acid chelates one Na^+^ cation with its O-donor of sulfonate group and of the carboxylate one, and chelates the other Na^+^ cation with another O-donor from the same sulfonate group and the amine N-donor. Fig.1c shows the coordination environment of the two Na^+^ cations: for Na1 it is five coordinated while for Na2 forms a 4 coordinated Na node. Moreover, Na1 and Na2 are bridged by one O atom from sulfonyl group. In conclusion, the Na cation and the L-Cysteic acid ligand construct a three-dimensional frameword with space group P21/c, Fig.2.

### Experimental

The title complex was synthesized through solvent-thermal reaction by L-Cysteic acid with NaOH aqueous as following method: 84.58 mg (0.5 mmol) of L-Cysteic acid, 10 mL 0.5% NaOH aqueous, were added to a 20 ml Teflon vessel. The vessel was sealed and placed inside a stainless steel autoclave, which was kept at 140°C for 72 h. Then the crystal suiting for X-ray single-crystal analysis was obtained.

### Refinement

H atoms bonded to N atom were located in a difference map and refined with distance restraints of N—H = 0.899–0.900 Å, and with *U*_iso_(H) = 1.2Ueq(N). Other H atoms were positioned geometrically and refined using a riding model, with C—H = 0.970–0.981 Å and with *U*_iso_(H) = 1.2 times *U*_eq_(C).

### Crystal data

**Table:**

[Na_2_(C_3_H_5_NO_5_S)]	*F*(000) = 432
*M**_r_* = 213.13	*D*_x_ = 1.875 Mg m^−^^3^
Monoclinic, *P*2_1_/*c*	Mo *K*α radiation, λ = 0.71073 Å
Hall symbol: -P 2ybc	Cell parameters from 7309 reflections
*a* = 5.7574 (12) Å	θ = 3.4–27.5°
*b* = 11.875 (2) Å	µ = 0.52 mm^−^^1^
*c* = 11.691 (3) Å	*T* = 298 K
β = 109.15 (3)°	Prism, colourless
*V* = 755.1 (3) Å^3^	0.24 × 0.22 × 0.20 mm
*Z* = 4	

### Data collection

**Table:**

Rigaku SCX-MINI diffractometer	1740 independent reflections
Radiation source: fine-focus sealed tube, Rigaku SCX-MINI	1463 reflections with *I* > 2σ(*I*)
graphite	*R*_int_ = 0.044
ω scans	θ_max_ = 27.5°, θ_min_ = 3.4°
Absorption correction: multi-scan (*ABSCOR*; Higashi, 1995)	*h* = −7→7
*T*_min_ = 0.885, *T*_max_ = 0.903	*k* = −15→15
7845 measured reflections	*l* = −15→15

### Refinement

**Table:**

Refinement on *F*^2^	Primary atom site location: structure-invariant direct methods
Least-squares matrix: full	Secondary atom site location: difference Fourier map
*R*[*F*^2^ > 2σ(*F*^2^)] = 0.060	Hydrogen site location: inferred from neighbouring sites
*wR*(*F*^2^) = 0.159	H-atom parameters constrained
*S* = 1.06	*w* = 1/[σ^2^(*F*_o_^2^) + (0.0698*P*)^2^ + 2.942*P*] where *P* = (*F*_o_^2^ + 2*F*_c_^2^)/3
1740 reflections	(Δ/σ)_max_ < 0.001
109 parameters	Δρ_max_ = 0.82 e Å^−^^3^
0 restraints	Δρ_min_ = −0.94 e Å^−^^3^

### Special details

**Table:**

Geometry. All e.s.d.'s (except the e.s.d. in the dihedral angle between two l.s. planes) are estimated using the full covariance matrix. The cell e.s.d.'s are taken into account individually in the estimation of e.s.d.'s in distances, angles and torsion angles; correlations between e.s.d.'s in cell parameters are only used when they are defined by crystal symmetry. An approximate (isotropic) treatment of cell e.s.d.'s is used for estimating e.s.d.'s involving l.s. planes.
Refinement. Refinement of *F*^2^ against ALL reflections. The weighted *R*-factor *wR* and goodness of fit *S* are based on *F*^2^, conventional *R*-factors *R* are based on *F*, with *F* set to zero for negative *F*^2^. The threshold expression of *F*^2^ > σ(*F*^2^) is used only for calculating *R*-factors(gt) *etc*. and is not relevant to the choice of reflections for refinement. *R*-factors based on *F*^2^ are statistically about twice as large as those based on *F*, and *R*- factors based on ALL data will be even larger.

### Fractional atomic coordinates and isotropic or equivalent isotropic displacement parameters (Å^2^)

**Table:**

	*x*	*y*	*z*	*U*_iso_*/*U*_eq_	
Na2	0.8052 (6)	0.7652 (3)	0.4635 (3)	0.0831 (9)	
Na1	0.0476 (3)	0.74867 (13)	0.32535 (13)	0.0217 (4)	
C1	0.2111 (7)	1.0060 (3)	0.3139 (3)	0.0182 (7)	
C2	0.4308 (6)	1.0333 (3)	0.2716 (3)	0.0172 (7)	
H2	0.4404	1.1156	0.2680	0.021*	
C3	0.3963 (7)	0.9894 (3)	0.1434 (3)	0.0193 (7)	
H3A	0.5202	1.0242	0.1154	0.023*	
H3B	0.2372	1.0147	0.0904	0.023*	
N1	0.6663 (6)	0.9944 (3)	0.3610 (3)	0.0220 (7)	
H1A	0.7827	1.0134	0.3287	0.026*	
H1B	0.6904	1.0396	0.4257	0.026*	
O1	0.2379 (5)	0.9342 (2)	0.3959 (2)	0.0237 (6)	
O2	0.0199 (5)	1.0610 (2)	0.2616 (3)	0.0286 (7)	
O3	0.2320 (6)	0.7884 (3)	0.1716 (3)	0.0304 (7)	
O4	0.6639 (5)	0.8093 (2)	0.1942 (3)	0.0293 (7)	
O5	0.3581 (5)	0.8235 (2)	−0.0035 (2)	0.0246 (6)	
S1	0.41314 (16)	0.84073 (7)	0.12588 (8)	0.0166 (3)	

### Atomic displacement parameters (Å^2^)

**Table:**

	*U*^11^	*U*^22^	*U*^33^	*U*^12^	*U*^13^	*U*^23^
Na2	0.081 (2)	0.097 (2)	0.0771 (19)	0.0052 (17)	0.0339 (16)	0.0134 (17)
Na1	0.0211 (8)	0.0228 (8)	0.0202 (8)	0.0001 (6)	0.0056 (6)	0.0015 (6)
C1	0.0185 (17)	0.0158 (17)	0.0214 (18)	−0.0033 (14)	0.0080 (14)	−0.0051 (14)
C2	0.0168 (16)	0.0161 (17)	0.0210 (17)	0.0001 (13)	0.0095 (14)	0.0002 (13)
C3	0.0240 (18)	0.0179 (17)	0.0187 (17)	0.0005 (14)	0.0104 (15)	0.0002 (14)
N1	0.0144 (14)	0.0333 (18)	0.0184 (15)	−0.0015 (13)	0.0054 (12)	−0.0058 (13)
O1	0.0263 (14)	0.0257 (14)	0.0208 (13)	−0.0038 (11)	0.0102 (11)	0.0012 (11)
O2	0.0187 (13)	0.0296 (15)	0.0399 (17)	0.0049 (11)	0.0128 (12)	0.0082 (13)
O3	0.0358 (17)	0.0297 (16)	0.0319 (16)	−0.0108 (13)	0.0195 (14)	−0.0023 (13)
O4	0.0251 (15)	0.0282 (15)	0.0276 (15)	0.0067 (12)	−0.0010 (12)	−0.0012 (12)
O5	0.0268 (14)	0.0298 (15)	0.0180 (13)	−0.0052 (11)	0.0084 (11)	−0.0049 (11)
S1	0.0180 (4)	0.0170 (4)	0.0151 (4)	−0.0012 (3)	0.0059 (3)	−0.0006 (3)

### Geometric parameters (Å, °)

**Table:**

Na1—O1	2.478 (3)	C2—N1	1.488 (5)
Na1—O2^i^	2.427 (3)	C2—C3	1.538 (5)
Na1—O3	2.415 (4)	C2—H2	0.9800
Na1—O4^ii^	2.351 (4)	C3—S1	1.783 (4)
Na1—O5^iii^	2.364 (3)	C3—H3A	0.9700
Na2—N1	2.976 (5)	C3—H3B	0.9700
Na2—O3^iv^	2.905 (5)	N1—H1A	0.9000
Na2—O4	3.028 (5)	N1—H1B	0.9000
Na2—O5^iii^	2.922 (5)	O3—S1	1.457 (3)
C1—O2	1.253 (5)	O4—S1	1.451 (3)
C1—O1	1.254 (5)	O5—S1	1.455 (3)
C1—C2	1.537 (5)		
			
O1—Na1—O5^iii^	84.93 (10)	C1—C2—H2	106.7
O1—Na1—O3	79.62 (12)	C3—C2—H2	106.7
O3—Na1—O4^ii^	90.18 (13)	C2—C3—S1	117.0 (3)
O4^ii^—Na1—O5^iii^	162.00 (13)	C2—C3—H3A	108.1
O3^iv^—Na2—N1	125.41 (16)	S1—C3—H3A	108.1
O3^iv^—Na2—O4	140.68 (16)	C2—C3—H3B	108.1
O3^iv^—Na2—O5^iii^	110.50 (14)	S1—C3—H3B	108.1
O4—Na2—N1	58.54 (11)	H3A—C3—H3B	107.3
O4—Na2—O5^iii^	104.59 (13)	C2—N1—H1A	105.0
O5^iii^—Na2—N1	104.53 (14)	C2—N1—H1B	105.0
O2—C1—O1	126.6 (3)	H1A—N1—H1B	105.9
O2—C1—C2	114.6 (3)	O4—S1—O5	111.96 (17)
O1—C1—C2	118.8 (3)	O4—S1—O3	113.02 (19)
N1—C2—C1	111.4 (3)	O5—S1—O3	112.53 (17)
N1—C2—C3	112.1 (3)	O4—S1—C3	105.86 (18)
C1—C2—C3	112.7 (3)	O5—S1—C3	104.89 (17)
N1—C2—H2	106.7	O3—S1—C3	107.93 (17)

Symmetry codes: (i) −*x*, *y*−1/2, −*z*+1/2; (ii) *x*−1, *y*, *z*; (iii) *x*, −*y*+3/2, *z*+1/2; (iv) *x*+1, −*y*+3/2, *z*+1/2.
